# Automating Digital Leaf Measurement: The Tooth, the Whole Tooth, and Nothing but the Tooth

**DOI:** 10.1371/journal.pone.0042112

**Published:** 2012-08-01

**Authors:** David P. A. Corney, H. Lilian Tang, Jonathan Y. Clark, Yin Hu, Jing Jin

**Affiliations:** Department of Computing, University of Surrey, Guildford, United Kingdom; Institut de Biologia Evolutiva - Universitat Pompeu Fabra, Spain

## Abstract

Many species of plants produce leaves with distinct teeth around their margins. The presence and nature of these teeth can often help botanists to identify species. Moreover, it has long been known that more species native to colder regions have teeth than species native to warmer regions. It has therefore been suggested that fossilized remains of leaves can be used as a proxy for ancient climate reconstruction. Similar studies on living plants can help our understanding of the relationships. The required analysis of leaves typically involves considerable manual effort, which in practice limits the number of leaves that are analyzed, potentially reducing the power of the results. In this work, we describe a novel algorithm to automate the marginal tooth analysis of leaves found in digital images. We demonstrate our methods on a large set of images of whole herbarium specimens collected from *Tilia* trees (also known as lime, linden or basswood). We chose the genus *Tilia* as its constituent species have toothed leaves of varied size and shape. In a previous study we extracted 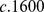 leaves automatically from a set of 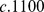 images. Our new algorithm locates teeth on the margins of such leaves and extracts features such as each tooth’s area, perimeter and internal angles, as well as counting them. We evaluate an implementation of our algorithm’s performance against a manually analyzed subset of the images. We found that the algorithm achieves an accuracy of 85% for counting teeth and 75% for estimating tooth area. We also demonstrate that the automatically extracted features are sufficient to identify different species of *Tilia* using a simple linear discriminant analysis, and that the features relating to teeth are the most useful.

## Introduction

Characterizing the margin of leaves, including their teeth, is important for several areas of botanical research. These include modeling the climate and identifying species, both of which we discuss here.

It was observed by Bailey and Sinnott in 1915–16 [1], [2] that in warm climates a greater proportion of plant species produce leaves with entire margins (i.e. smooth leaf edges without teeth) and fewer produce dentate or serrate leaves (i.e. with teeth on their margins). Conversely, in cool climates more species tend to produce toothed leaves. Due to the presence of a large number of well-preserved leaves in the fossil record, it has also been proposed that the morphology of fossilized leaves can be used as a “paleothermometer” to aid in modeling past climates [3]–[8].

This estimation of temperature by calculating the proportion of toothed species at a site is called “leaf margin analysis” (LMA) [3], [5]. In its standard form, this is a very simple univariate model predicting mean annual temperature from the proportion of species with entire (toothless) margins. This relationship has been empirically shown to hold for a range of temperate and tropical forests of dicotyledonous trees although it becomes less accurate for very cold, dry regions. Note that the term “blade” refers strictly to the flat part of a leaf, while “leaf” refers to both the blade and the petiole (stalk); in this work, we are only interested in the blade and its margin [9].

In this classical LMA, each species is given a binary value simply recording the presence or absence of teeth. There have been attempts to produce more accurate models by introducing more variables and characterizing the leaf margins using more sophisticated measures than simply the presence or absence of teeth. For example, CLAMP [4] included scores for the presence of lobes and teeth, seven different leaf size classes and three leaf shape classes. Such characters are still limited to having only two (or a few) valid states, as opposed to continuous measurements of size, shape etc. Also, the character states are defined ambiguously, meaning that different researchers interpret them differently. [5], [10].

One more recent approach is “digital leaf physiognomy” [6]–[8], a rigorous method of analyzing the size and shape of leaves, both fresh and fossilized. This extends the classical LMA approach in several ways, including the incorporation of continuous variables. For example, it includes a count of the number of teeth present, rather than just a binary present/absent indication, along with blade area, perimeter, length and width. Some studies have also specifically included measures of tooth area and blade area and their ratio [11], as we do here. Huff, Wilf and Azumah [7] collected 283 leaves from three distinct living floras (two temperate forests and one tropical moist forest). Using Adobe Photoshop 6.0, they touched up each image to restore obviously damaged sections, then manually selected every tooth and separated them from the rest of the lamina. They then calculated the area of the whole leaf lamina and the area of the separated teeth. A more recent study by Peppe et al. [8] uses the same methods on a much larger scale, analyzing over 6500 leaves from 92 diverse sites (including tropical rainforests, temperate forests, shrubland, and desert) as well as analyzing 10 fossil floras, and used ImageJ software (http://rsb.info.nih.gov/ij) to estimate teeth area. This required a great deal of manual effort to first identify the boundary of every tooth. Related work has measured the change in leaf shape along temperature gradients [12]. In this paper, we describe software that automates this process and we compare this against manual measurements replicating the digital leaf physiognomy methods.

In both digital leaf physiognomy studies mentioned above, the analysis was repeated manually for every leaf, a process the authors acknowledge to be “labor intensive” [7] and to risk introducing human error. They state that “no existing software can discriminate teeth from leaf lamina with sufficient reliability” [7]. They also used the major axis length as a proxy for leaf (blade) length, arguing that this latter “must be measured manually”. While major axis length is highly correlated with blade length, they acknowledge that some leaves are wider than they are long, which will result in misleading length estimates. To overcome this, they typically calculate the ratio of major axis to minor axis (the length to width ratio) whereas we use estimates of the blade length and width as defined in standard botanical works [9], [13]. Also, some leaves, including those of some *Tilia* species, can be asymmetric to the extent that the major axis is somewhat displaced or rotated from the usual measurement position of length. Our work goes some way to addressing these gaps by developing software that can find, count and characterize teeth automatically to complement our recent work [13] that automatically measures the length of a leaf’s lamina.

Although the function of marginal teeth is still a matter of debate [8], one theory suggested by Royer and Wilf is that teeth increase gaseous exchange and allow more photosynthesis, especially during the early growing season [14]. They also suggest that the surface area:volume ratio will be highest at the edges of a leaf, and raised further by the presence of teeth. Larger surface area:volume ratios will allow improved gaseous exchange, and therefore photosynthesis and growth, especially in the early growing season in cold, wet climates. This increased gaseous exchange occurs alongside increased sap flow [2], [4], [14]. In such climates, there is also a risk of leaves becoming over-saturated with water, filling intercellular air spaces with water and so reducing the rate of CO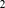 absorption and therefore of photosynthesis [15]. In warmer climates, these benefits may be outweighed by the cost of increased water-loss [8].

Besides climate modeling, the counting and characterizing of leaf margin teeth can also play a part in species identification. For example, Pigott [16] provides a formal taxonomic description of cultivated species of *Tilia*, the genus we use in our experiments here, and provides a taxonomic key (a branching set of rules used to identify species). When distinguishing certain species, he includes descriptions of teeth size and/or variation in size as relevant features. For example, part of Pigott’s key relating to *T. japonica* includes “uniform triangular teeth less than 1.5 mm wide and long…” while the part of the key relating to *T. mongolica* includes “leaf-margins with a few large teeth…”. The leaves we analyze in this paper are from specimens stored at the herbarium of the Royal Botanic Gardens, Kew. They were collected from 18 species of *Tilia*; further details of these specimens are given by Corney et al. [13] A broader review of the classification of the *Tilia* genus is provided by Clark [17]; see also Pigott’s forthcoming book [18]. In addition, a general review of species identification using images of botanical specimens has recently been published [19].

Identifying species from images of leaves, flowers or other plant organs is challenging for a number of reasons. Such structures are typically non-rigid and three-dimensional, which means that consistent, high-quality images are hard to generate. Furthermore, as the plant material dries out, it typically changes color, texture and shape, and these can continue to change for some time after collection. Leaf shape can vary considerably not only between different specimens of the same species, but also between different leaves on the same plant. Some plants exhibit “heteroblasty”, where leaf shape varies along a single stem as the leaves develop. These and other issues are discussed in detail in [19].

One of the more successful general-purpose plant species identification systems is “LeafSnap” [20], which runs on a mobile phone and attempts to recognize species from images. However, this system is limited to the range of plants found at three sites in the United States. It can recognize a few hundred species. Estimates of numbers of species of flowering plants (or angiosperms) vary from about 220,000 [21] to 420,000 [22].

**Figure 1:**
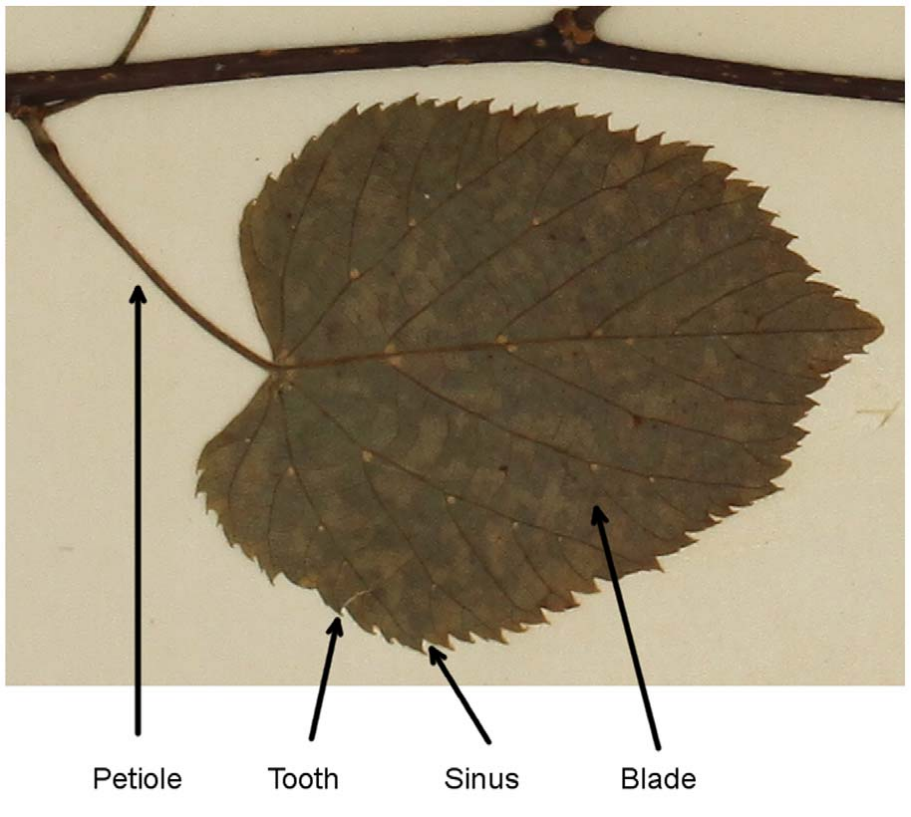
A detail from a typical herbarium sheet. In this example, the leaves are held in place with glue, which are seen here as translucent blobs. Other non-leaf objects appear, such as the stalk at the top. The specimen is mounted on heavy cartridge paper (c. 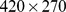 mm).

Figure 1 shows detail from a typical herbarium specimen. The leaf of interest has a toothed margin, and appears relatively undamaged. In many cases, such leaves are partly hidden behind other leaves or non-leaf objects, and often also show damage from tearing or from herbivores. This makes automated image analysis more challenging than it is for fresh, undamaged leaves, but digitized herbarium specimens are available in large numbers and are botanically important. Collectively, the world’s major herbaria contain over 350 million specimens [23] that have been collected from around the world for over 250 years, and many of these are in the process of being digitized. In this work, we will be analyzing herbarium specimens rather than fresh or fossilized leaves.

Having established that is it useful for botanists to count and characterize any teeth present on a leaf margin, let us consider three options for measuring teeth.

### Manual

Leaves can be examined by hand either in the field or in herbaria. This approach is accurate for counting teeth, although rather slow and labor intensive. It is also hard to estimate tooth area or perimeter however, which is perhaps why such characters are rarely used in traditional taxonomic keys for species identification. Although accurate, it is worth noting that people are not perfect at counting large numbers of objects, so repeated trials are likely to generate slightly different results, even if great care is taken. When measuring leaf area, it has been found that measurement errors account for less than 0.1% of the total variation [24], the main variation being due to variability of leaves within a single tree and among populations.

### Digitized

If digital images of leaves are created and examined, then tools such as ImageJ can be used to measure tooth area and related features. This still requires great manual effort, to select the boundary of each tooth in a graphics package, for example, which limits possibilities. There will still be some variation between different people performing the same task, as different users will inevitably draw slightly different boundaries or make different decisions when selecting teeth.

### Automated

If specially-written software is used that automatically analyzes digital images, with minimal human effort required, then arbitrarily large sets of leaves can be analyzed. Furthermore, software can measure features such as area and angle as well as counting teeth. The cost to this approach is that software tends to fail to reach human performance levels at tasks such as those involving pattern recognition and image processing. Thus, as in many other areas, there is an inevitable trade-off between automation and accuracy.

The main purpose of the algorithm described here is to automate a task that is currently carried out in a manual and/or digitized (but not automated) fashion, and thus to increase the value of specimens in herbaria for studies of morphological variation. With such automation, there is always a trade-off between a gain in speed and a loss of precision. The automated system we describe here may be less accurate than the manual approach, but it requires minimal human intervention and only readily-available computer resources. The main goal is speed: once extracted, thousands of leaf images can be processed in less than an hour, allowing for far larger studies than would otherwise be practical or possible.

One existing system for leaf analysis is the WinFOLIA software tool (http://www.regentinstruments.com/products/folia/FOLIA.html). This is a commercial tool that can be used as a portable leaf area meter, and is capable of counting teeth and measuring tooth area, along with a number of other botanically relevant features. It is not clear how successful this would be for analyzing herbarium specimens, as opposed to single-leaf images, but it does include tools to allow the user to manually separate the leaf from background clutter, and to manually select and “restore” damaged parts of the leaf.

We recently demonstrated a method for automated character extraction from whole herbarium specimens [13]. In that work, we described software that automatically analyzed images to locate leaves and to find the exact boundary of those leaves. In brief, that software used deformable templates, optimized by evolutionary computing algorithms, to quickly find the approximate location of each leaf in the image. This approximation was then improved using a level-set method to find the exact boundary separating the leaf from the background (plain paper) and from other objects present (such as other leaves, stems, flowers). Having found and recorded the outline of each leaf, that system then used local morphological characteristics to identify the primary vein and then automatically extracted the length and width of each leaf blade. With minimal human effort, this system analyzed 1127 images and located and measured 1645 leaves. Although the results were not perfect, the quality was high enough to obtain a strong correlation between the extracted leaf blade dimensions and similar measurements reported in the literature.

Here, we extend that work and describe a new algorithm that analyzes the leaf outlines that have been extracted from the whole herbarium specimens. We use local morphological features to locate any teeth present on the margin of the leaf lamina. Once located, we represent each tooth as a triangle and measure its area, perimeter, internal angles and other features as described below. We compare these automatically extracted characters with a manually measured sample, and demonstrate their value in a simple species identification task. We have limited our studies here to images of herbarium specimens, and not experimented with either a) images of isolated fresh leaves nor b) images of fossils. We believe that compared to the current work, the first of these is likely to yield better results, as the leaves are undamaged, while the latter is likely to yield worse results, as fossils are often fragmented. While further research is necessary, we believe that our methods are a useful contribution to the area.

## Methods

We now describe our algorithm and two sets of experiments to evaluate it. We compare the algorithm’s estimates of tooth count and tooth area with manual estimates and then use the extracted characters to perform basic species identification.

### Tooth-finding Algorithm Details

In previous work [13], we described a method for finding the outline of leaves in digital images of whole herbarium specimens. Such outlines can also be found from images of isolated leaves on plain backgrounds using straightforward segmentation methods (e.g. [19], [25]). In this work, we use the outlines found by our previous software, though all our methods require is that the edges of the leaves are represented as an ordered set of 2D Cartesian co-ordinates. We do not assume that the outline is “perfect”, nor that the leaf itself is free from damage. Instead, we attempt to locate and characterize any teeth that are present and do not attempt to infer the characteristics of any “missing” teeth. Here, we provide details of our algorithm for finding and characterizing marginal teeth, given a leaf outline.

**Figure 2:**
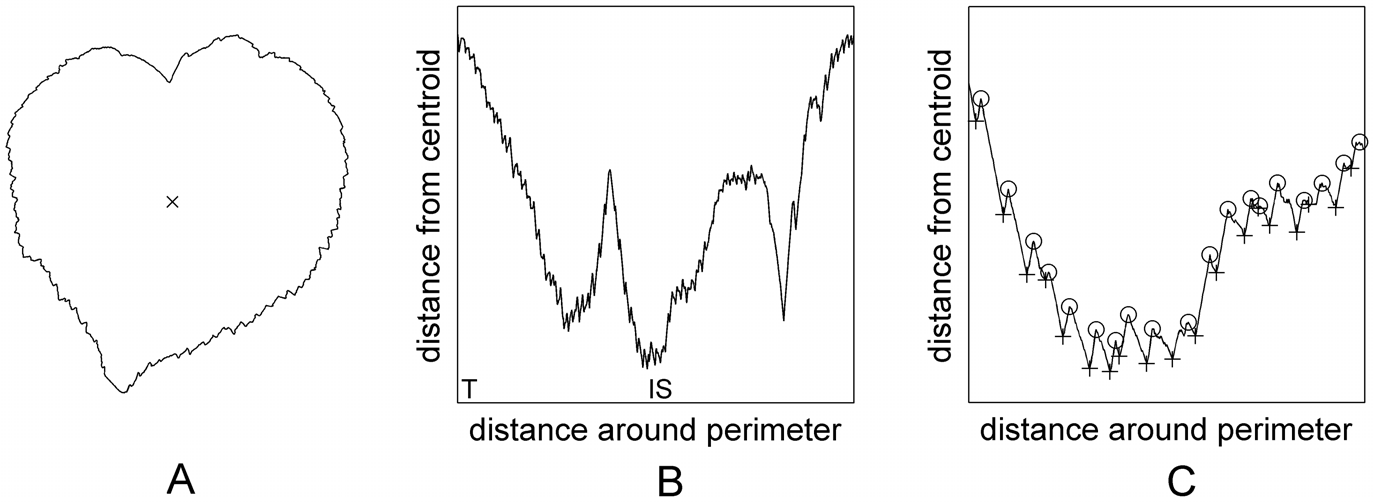
Centroid-contour plots. A) The outline of a single leaf with the centroid marked. B) The centroid-contour plot for the same leaf. The left side of the plot corresponds to the leaf tip (marked ‘T’) and moves clockwise around the leaf through the insertion point at the top of the leaf (marked ‘IS’) and back to the tip at the right hand side. C) A detail of the centroid-contour plot around the insertion point. Here, individual teeth tips are marked by our software with a circle and each sinus is marked with a cross.

For each leaf outline, we first find the centroid of the leaf by calculating the mean of all the edge points (Figure 2A). We then calculate the distance from the centroid to each edge point in turn (Figure 2B). We then analyze the local morphology to identify teeth by calculating the change in the distance to the centroid. Intuitively, the tip of a tooth will be represented as a local maximum of this function, while a sinus between two teeth will be represented by a local minimum. By taking the first numerical difference of the distance vector, we can find all such local extrema (Figure 2C). Table 1 provides an outline of the main algorithm. Note that we count all teeth present and do not distinguish between primary and secondary teeth.

**Table 1:** Outline of algorithm for tooth finding algorithm.

Input: a set of coordinates 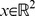
*teeth* 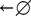
*sinuses* 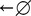
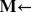 centroid 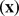
# Calculate distance from centroid to contour:
for 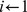 to 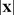
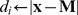
# Identify probable teeth and sinuses
for 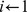 to 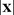
if 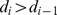 and 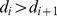 then add 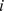 to *teeth*
if 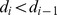 and 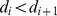 then add 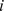 to *sinuses*
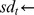 standard deviation of 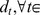 *teeth*
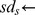 standard deviation of 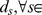 *sinuses*
# Remove excessively large (or small) teeth from final set
for 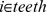
if 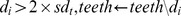 # remove unusually large objects
if 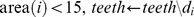 # remove very small objects
if 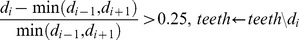 # remove lobes
Output: *teeth, sinuses*

Pseudocode for tooth finding algorithm. Teeth are initially defined as locally maximum centroid-contour distances. Very large objects, very small objects and lobes (with incision fractions greater than 25%) are then removed from this set.

Let 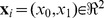 be the 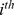 point on the margin of a leaf, defined by the image co-ordinates 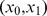. Then the centroid 
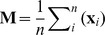
 where 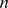 is the total number of points on the edge. The distance from each marginal point to the centroid is 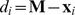. As an initial approximation, where 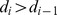 and 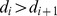, we label 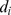 as the tip of a tooth and where 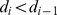 and 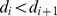, we label 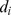 as a sinus. In this simplest of forms, the method will often detect minor defects in the leaf and falsely label them as teeth; to reduce this, we ignore very small local deviations, and only label a point as a tooth if it has a distinct sinus on both sides. We also reject all candidate teeth that are much larger than average to remove incorrectly identified regions. Specifically, we initially calculate the standard deviation of the areas of all the candidate teeth, and then reject anything that is more than two standard deviations above the mean. This often occurs where leaves have been damaged, because a torn edge can resemble a serrated margin. Such a correction should also help to distinguish teeth from lobes, although the *Tilia* specimens that we are analyzing here do not tend to be lobate. The Manual of Leaf Architecture [9] distinguishes a lobe from a tooth by considering the depth of the incision. A tooth is a “projection separated by sinuses that are incised less than 25% of the distance to the midvein” [p. 28]. If the incision on either side is more than 25%, the projection is considered to be a lobe [p. 26]. We incorporate this threshold to reject lobes and not count them as teeth. As described by Royer et al. ([6], Appendix S1), a more reliable test to distinguish lobes from teeth involves measuring the distance from the sinus to the midvein along the axis of symmetry of the indentation. We use the shortest distance from the sinus to the leaf centroid as an approximation to this.

**Figure 3:**
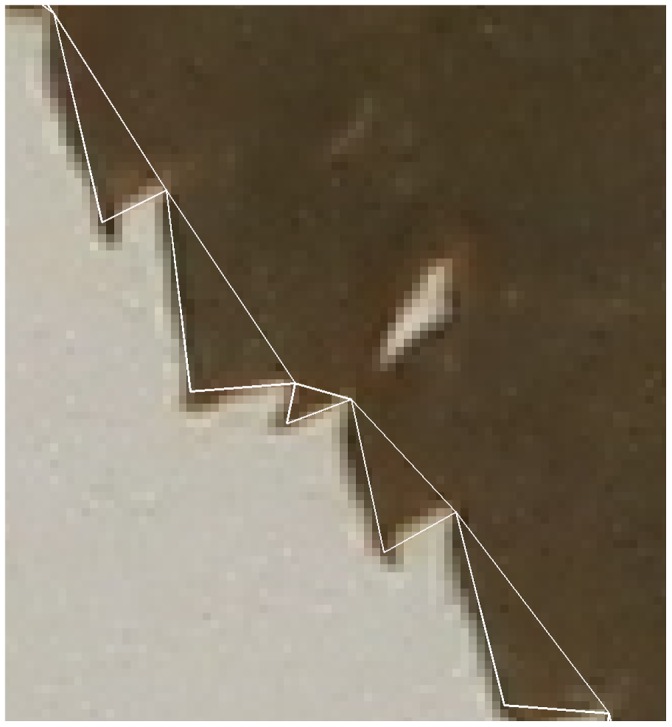
Detail showing several teeth from the margin of a leaf. Each tooth is approximated with a triangle.

**Figure 4:**
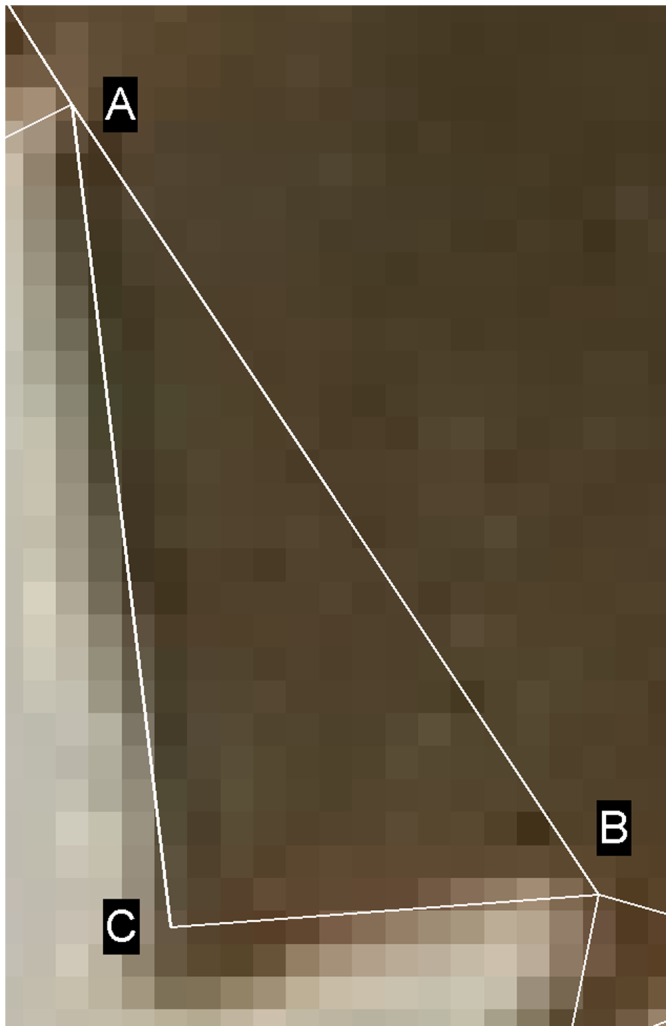
A single tooth modeled as a triangle. The visible pixelation is due to this being a small detail from a much larger image.

Each tooth we find is defined by three points: the tip of the tooth and the sinus to either side. Figure 3 shows five teeth from the margin of one leaf, each represented by a white triangle. Figure 4 shows one of these teeth in detail, with the two sinuses labeled A and B, and the tip labeled C. For simplicity, we treat the tooth as a triangle and ignore the (typically very slight) deviations from straight lines found in the sides of each tooth. Treating the tooth as a triangle makes it trivial to calculate the area and the angle between two outer edges of the tooth.

We model the 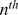 tooth as a triangle A
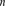
B
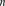
C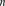. We use the convention that 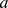 is the length of the side opposite corner A and so on, so tooth 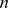 has a total outer edge of length 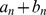. We also define the base of the triangle (AB) as the implicit boundary between the tooth and the rest of the lamina, which has a length of 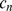. For each leaf, the software counts the number of teeth, the total area of all the teeth, the angles at the tips of the teeth and the inner perimeter. From these values, we can also derive features such as the “frequency” of teeth (i.e. the number of teeth per unit length of margin), the mean tooth area, and so on. A full list of all the features used in this paper is given in Table 2.

**Table 2:** List of automatically extracted features.

Blade length	*L*
Blade width	*W*
*Number of teeth	*N*
Total blade area	S
Total blade perimeter	*P*
*Total length of outer edges	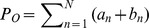
*Total tooth base length	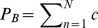
*Inner perimeter (i.e. perimeter of blade after teeth are removed)	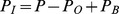
*Total area of teeth	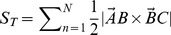
*Blade area excluding teeth	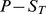
Compactness	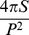
Shape factor	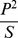
*Mean angle at tip	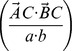 arccos 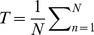
*Frequency of teeth	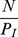
*Relative tooth area	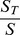
*Mean ratio of lengths of outer edges	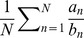
Perimeter Ratio	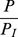
*Tooth Area Blade Ratio	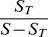
*Tooth Num Blade Ratio	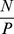
*Mean Tooth Area	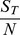

List of extracted features. The right-hand column gives the definitions in terms of the points A and B (the two sinuses) and C (the tooth tip), as in Figure 4, with 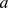 being the length of BC, 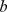 the length of AC and 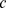 the length of 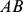. The form 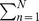 refers to summing a value across all the identified teeth.

* Tooth-related characters. Features without a star relate to the blade but not specifically the teeth. Some of the experiments here distinguish between “tooth related” and “non-tooth related” characters.

We have implemented the algorithm using Matlab v.7.10 (MathWorks, Natick, MA, USA) on a standard desktop PC (3.1 GHz CPU, 4 Gb RAM). Processing a single leaf takes around 0.8 s. This could undoubtedly be improved through further optimization and/or using parallel computing. The source code is available via http://www.computing.surrey.ac.uk/morphidas/.

### Measuring Teeth Manually

In order to establish ground truth values and to evaluate the software, we chose 50 leaves at random for a manual analysis. In a few cases (six), the leaf extraction method described previously [13] failed to work satisfactorily, and the automatically extracted leaves were either very badly damaged, substantially hidden behind other leaves, or had in some way been incorrectly extracted. To include these images in the evaluation would mean penalising the tooth-finding algorithm for mistakes made elsewhere. We removed these examples and replaced them with different randomly chosen leaves before carrying out the comparisons.

We followed previously-published methods for the manual analysis [6]–[8]. Accordingly, we used the open-source image editor GIMP (www.gimp.org/) to edit the image of each leaf, cutting each tooth in turn and pasting it into a second image file, counting the teeth in the process. We then loaded this latter file into ImageJ v.1.42q (National Institute of Mental Health, Bethesda, Maryland, USA; http://rsb.info.nih.gov/ij), and used the “Analyze particles” routine to calculate the number and area of the teeth. The whole process took in the order of 20 minutes per leaf after initial training, with most of time consumed in selecting individual teeth. We do not count the very tip of the leaf as a tooth, as this area includes the primary vein [7]. The tip is often 10 or 20 times larger than the teeth of the same leaf. Including this as a tooth would distort measures such as the mean tooth size, total tooth area and so on.

Note that the area calculated by ImageJ is based on the number of pixels forming each tooth and unlike our software, it does not assume that each tooth is triangular. The ImageJ results therefore incorporate the typical slight convexity of teeth, which could lead to a small discrepancy in the results compared to our software’s output.

We measure the accuracy of counting the teeth using the relative error. If the software found 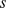 teeth and the manual inspection found 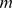 teeth, then the relative error is 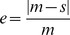. Similarly for the area, we calculated the relative error based on the difference between the software’s estimate of total area and the manual calculations (via ImageJ). These relative areas are unitless and allow us to compare errors relating to leaves with widely varying characters. We also calculate the root-mean-square (RMS) error, which has the same units as the features being measured. We assume that the manual measurements are perfect, though we acknowledge that some errors are inevitable. For comparison, Huff et al. [7] estimated the error in their manual area measurements as less than 1 mm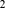, although no details are given.

**Table 3:** Summary of results.

Tooth count				
*n*	Manual	Software	Relative error	RMS error
50 leaves	51.52	54.24	0.1496	8.766
**Total tooth area**				
*n*	**Manual**	**Software**	**Relative error**	**RMS error**
951 teeth( = 16 leaves)	75.96	66.79	0.2480	24.01

Summary of results, comparing the manual estimates of the mean number of teeth per leaf and the total area of the teeth per leaf, with the software’s estimates of the same measures. The mean relative absolute error and root-mean-square error are also given.

### Classification Methods

To test the effectiveness of automatic character extraction, we carried out some simple species identification experiments. The main goal here is to test the usefulness of the automatically extracted tooth-related features, rather than to produce the optimum classifier. We therefore only use a subset of the data and a simple classifier, as we discuss below. Note that the term “classification” is used in taxonomy to refer to the grouping of items by similarity. Here, we use the word in the statistical and machine learning sense: the assigning of an item to one of a fixed set of pre-defined classes. In taxonomy, this is often called identification, or in this case, species identification.

We use linear discriminant analysis (LDA) as this simple method provides a useful benchmark. The results are likely to be improved by using more sophisticated non-linear algorithms such as support vector machines or artificial neural networks, as we describe in related ongoing work [26]. The LDA method works by estimating the parameters of a Gaussian distribution for each class being modeled. For each previously unseen test point, the probability of it having been generated by each distribution is calculated and the class with the highest probability is assigned to that point. To provide an unbiased estimate of the model accuracy, we need to divide the data into a non-overlapping training set (90% of the records), used to estimate the model parameters, and a testing set (10% of the records), used to estimate its accuracy.

We use LDA to predict the species labels using the extracted features. The classes used have widely ranging sizes, because different numbers of leaves were found for each specimen and because the number of available specimens varied widely between species. In a number of cases, very few leaves were found for some species. To mitigate these issues, in this part of the analysis we restrict the scope to the four species with the greatest number of available leaves, namely *Tilia cordata*, *T. americana*, *T. platyphyllos* and *T. tomentosa*. We balanced the data set by randomly sampling (without replacement) an equal number of examples from each species, limited by the size of the smallest class. We then used stratified sampling such that each training set contains (approximately) the same number of samples of each species under consideration. We also normalized the data so that each feature had a mean of zero and a standard deviation of one. To obtain reliable estimates of model accuracy we used 10-fold cross-validation, and repeated the whole process 100 times to allow for the effect of the randomized sampling. In each case, the model was evaluated using a hold-out test set of data that had not been used to build the model. Note that because the data sets are stratified, all classes (i.e. species) have equal prior probabilities.

We carry out two sets of studies. First, we use a single multi-class LDA to predict all four classes. This is a challenging task: requiring one model to model several classes simultaneously is always hard. Therefore we carried out a second set of studies using two-class LDA models. Here, one LDA model is trained to classify *T. cordata* and *T. americana*, a second trained to classify *T. cordata* and *T. platyphyllos*, and so on, with a total of six classifiers.

In each study, we analyze the usefulness of the tooth-related features by building classifiers using a) tooth features only; b) non-tooth features only; and c) both sets of features. The non-tooth features are leaf characters such as blade area and perimeter. The automatic extraction of these features is described in [13]. Table 2 lists both sets of features.

**Figure 5:**
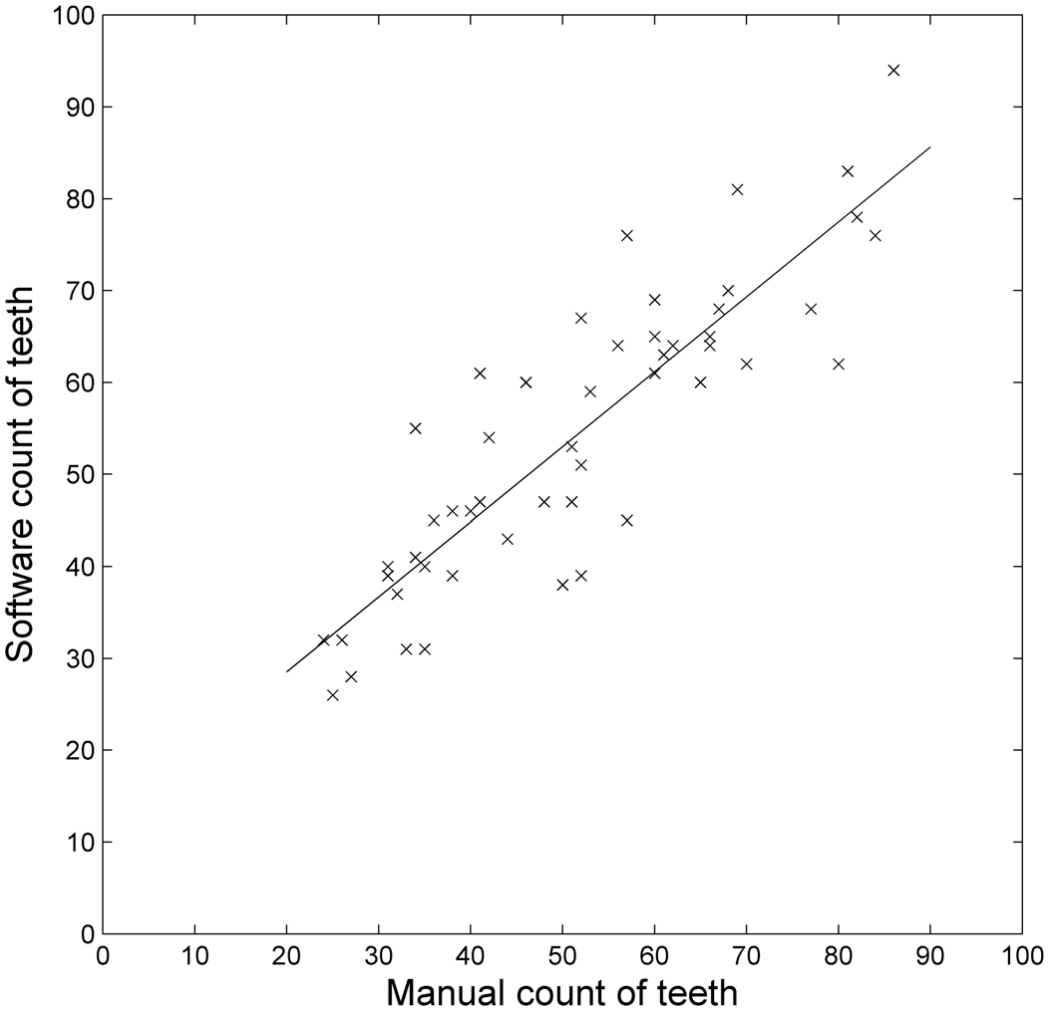
Comparison of manual count of teeth on each leaf with the system’s estimate. Each cross represents one of 50 leaves. The line is a least-squares best-fit line.

## Results

### Counting and Measuring Teeth

Here, we estimated the accuracy of the software’s feature extraction by comparing the output values to manual estimates described above. The results are summarized in Table 3.

**Table 4:** Species identification using multi-class LDA.

Features used	Mean accuracy	Standard deviation ofaccuracy
None (random classifier)	25.00	–
Non-tooth	35.38	1.69
Tooth	42.06	1.49
All	43.16	1.56

Comparison of LDA (linear discriminant analysis) classification accuracy using different sets of features. The “random classifier” assigns class labels at random, and scores 25% because there are four possible classes. The list of “tooth related” and “non-tooth related” features is given in Table 2. Two-tailed 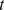-tests comparing each classifier’s accuracy with the classifier of the row above all showed significance with 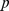-value 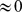. Results shown are based on 100 trials.

From the manual counts we found that this sample of 50 leaves had between 22 and 86 teeth each, with a mean of 51.52 teeth per leaf. The system predicted a mean of 54.24, showing only a very slight over-estimation on average. The correlation between the manual and software counts was 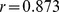 (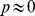), showing a very strong positive correlation. The root-mean-square error between the manual and software estimates was 8.76, suggesting that the predictions were typically accurate to within less than 9 teeth either way. In fact 78% of cases had single-digit errors, while the largest errors were an underestimate of 21 teeth and an overestimate of 18 teeth. A paired 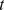-test shows a significant difference (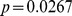), suggesting some systematic pattern to these errors despite the high overall accuracy. In this case, the mean relative error is 0.15, which is equivalent to an accuracy of 85%. Figure 5 compares the manual count (horizontal axis) with the software’s estimated teeth count (vertical axis) for all 50 leaves. The best-fit-line on the graph does not go through the origin, but has a 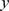-intercept of around 12. This shows that there is a small but systematic over-estimate of the number of teeth, as noted above. Thus although the software is not perfect, it does give a good indication of the number of teeth present across a wide range of values.

**Table 5:** Species identification using pairwise LDA.

Non-tooth features (mean accuracy: 60.7%)			
	*T. platyphyllos*	*T. americana*	*T. tomentosa*
*T. cordata*	60.61	66.20	61.35
*T. platyphyllos*		60.41	57.63
*T. americana*			57.78
**Tooth features (mean accuracy: 67.8%)**			
	***T. platyphyllos***	***T. americana***	***T. tomentosa***
*T. cordata*	66.26	77.80	72.16
*T. platyphyllos*		66.40	61.22
*T. americana*			62.56
**All features (mean accuracy: 68.3%)**			
	***T. platyphyllos***	***T. americana***	***T. tomentosa***
*T. cordata*	65.84	81.14	72.47
*T. platyphyllos*		67.00	59.72
*T. americana*			64.06

Comparison of LDA (linear discriminant analysis) classification accuracy of each pairwise model. The list of “tooth related” and “non-tooth related” features is given in Table 2. Two-tailed 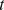-tests show that the mean accuracy of each of the three feature sets is significantly higher than the previous set, or significantly higher than a random classifier in the first case (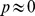 in all cases).

To complement this, we also manually measured the area of a number of teeth using the ImageJ application, following the methods described by Peppe et al. [8] and others. We manually measured the area of 951 teeth, specifically those visible in 16 leaves drawn from the random sample of 50 leaves used above. The total tooth area for a leaf was on average 76 mm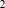, compared with the software’s estimated average of 67 mm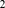. The mean relative error for total tooth area was 0.248, equivalent to an accuracy of over 75%, and a root-mean-squared error of 24 mm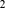. The worst case was an overestimate by 46 mm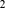, though most errors were 10 mm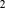 or less. A paired 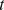-test shows no significant difference between the manual and automated estimates of area (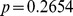).

### Species Identification

Having established the basic accuracy of our system, we now demonstrate the potential value of automated leaf margin character extraction, with some experiments in species identification (Table 4). For the multi-class LDA, we randomly sampled 131 records from each class as that is the size of the smallest class.

Given that there are four equal-sized classes, it is trivial that a purely random estimator would achieve an accuracy of around 25% true-positive. Using only non-tooth features, such as blade length and width, produces an LDA with a classification accuracy of just over 35%. A two-tailed 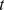-test shows that the LDA using only non-tooth features performs significantly better than the random classifer would do (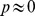). However, using the tooth features alone produces an accuracy of over 42%, also a significant increase. Combining tooth and non-tooth features gives a slight further improvement to 43%.

We then created a series of 6 binary classifiers, each trained to distinguish between two species. With just two balanced classes, a random classifier would be 50% accurate. The average accuracy of the 6 classifiers was 60.7% using non-tooth features, 67.8% using tooth-features and 68.3% using all features. Each of these successive improvements is significant according to two-tailed 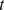-tests (with 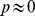 in each case). Table 5 show the relative performance of each pairwise classifier. It also shows that certain pairs of species are relatively hard to classify, leading to low accuracy scores. For example, *T. tomentosa* and *T. platyphyllos* are only distinguished with around 57–61% accuracy, while *T. cordata* and *T. americana* are distinguished with up to 81% accuracy.

These results clearly show that using the automatically extracted features allows identification of species at considerably better than chance rates. Furthermore, using tooth-related features significantly improves the results compared with using other features.

## Discussion

In this work, we have demonstrated that it is possible to automatically locate and measure leaf margin teeth from images of herbarium specimens, extending our previous work in this area [13]. We have also shown that after identifying teeth, it is possible to automatically extract characters from them such as tooth size and shape. As far as we are aware, this is the first time such automation of the analysis of leaf margins from herbarium images has been demonstrated. We believe that the accuracy is sufficient to demonstrate the potential benefits of automation in tasks such as climate modeling and species identification.

This paper is focused on describing a novel algorithm and performing some initial evaluation. We have therefore not attempted to also carry out detailed climate modeling using the extracted leaf features. One possible future study would be to use a set of specimens that have already been used to estimate climatic features, automate the character extraction, and build a similar model. The results could then be compared with the model built from manually-extracted characters.

Our results suggest a trade-off between speed and accuracy. One option is to use manual estimations, which are relatively slow but (allowing for slight human errors) highly accurate. The alternative is an automated system such as the one we present here, which reduces accuracy slightly but is significantly faster. The manual measurements we used here for purposes of comparison followed a similar procedure to that used in previous work [7], [8] and took around 20 minutes per leaf. The software, running on a standard desktop PC took less than a second per leaf, over 1000 times faster. This potentially allows far larger studies to be carried out when the images are available, facilitating studies of variation in species and populations. Ongoing digitization of herbarium specimens means that such images are already available in many cases.

The species classification experiments are included to demonstrate the usefulness of tooth features in identifying species: they allow pairwise classification with an accuracy of up to around 81% in this case. As noted above, the accuracy could be improved further in a number of ways, such as by using non-linear classifiers (e.g. artificial neural networks), improved features (e.g. more sophisticated modeling of tooth shape) or more sophisticated feature selection methods. In a closely related study, we demonstrated the use of artificial neural networks on equivalent data, and achieved marginally stronger results [26]. Multiclass classification is always a challenge, and it would be naïve to expect the methods we describe here to successfully scale up to distinguishing between thousands of species. Identifying species within a given genus is a more common task for taxonomists and it is that task that we have demonstrated here, albeit for only a subset of the species.

The method could also be applied to images of single leaves (rather than extracts from herbarium specimens). This is likely to improve the results, as single-leaf images tend to show leaves that have been carefully selected to be free from major damage, and free from obscuration or clutter from other objects in the image. Fossilized leaves vary widely in quality in this regard, with some being almost undamaged while others are reduced to fragments. This presents a substantial challenge for any automated process, but one that we feel is comparable to the challenges of analyzing herbarium images, as these also contain damaged, overlapping or incomplete leaves. In its current form, our method is not appropriate for automated fossil leaf analysis but it is, we believe, a step in that direction. We have only used our algorithm for leaves of the genus *Tilia*; it may need some adjustments to work as effectively with other genera, but the same approach should work for a wide variety of leaves.

## References

[1] Bailey IW, Sinnott EW (1915) A botanical index of Cretaceous and Tertiary climates. Science 41: 831–834. 17835989 10.1126/science.41.1066.831

[2] Bailey IW, Sinnott EW (1916) The climatic distribution of certain types of angiosperm leaves. American Journal of Botany 3: 24–39.

[3] Wolfe JA (1979) Temperature parameters of humid to mesic forests of eastern Asia and relation to forests of other regions in the Northern hemisphere and Australasia. United States Geological Survey Professional Paper 1106.

[4] Wolfe JA (1993) A method of obtaining climatic parameters from leaf assemblages, volume 2040 of *U.S. Geological Survey Bulletin* . U.S. Government Printing Office, 71 pp.

[5] Wilf P (1997) When are leaves good thermometers? A new case for Leaf Margin Analysis. Paleobiology 23: 373–390.

[6] Royer DL, Wilf P, Janesko DA, Kowalski EA, Dilcher DL (2005) Correlations of climate and plant ecology to leaf size and shape: potential proxies for the fossil record. American Journal of Botany 92: 1141–1151. 21646136 10.3732/ajb.92.7.1141

[7] Huff PM, Wilf P, Azumah EJ (2003) Digital future for paleoclimate estimation from fossil leaves? Preliminary results. Palaios 18: 266–274.

[8] Peppe DJ, Royer DL, Cariglino B, Oliver SY, Newman S, et al (2011) Sensitivity of leaf size and shape to climate: global patterns and paleoclimatic applications. New Phytologist 190: 724–739. 21294735 10.1111/j.1469-8137.2010.03615.x

[9] Ellis B, Daly DC, Hickey L, Johnson KR, Mitchell JD, et al (2009) Manual of leaf architecture. New York, USA: Cornell University Press.

[10] Green W (2006) Loosening the CLAMP: An exploratory graphical approach to the climate leaf analysis multivariate program. Palaeontologia Electronica 9: 9A.

[11] Royer DL, McElwain JC, Adams JM, Wilf P (2008) Sensitivity of leaf size and shape to climate within Acer rubrum and Quercus kelloggii. New Phytologist 179: 808–817. 18507771 10.1111/j.1469-8137.2008.02496.x

[12] Royer DL, Meyerson LA, Robertson KM, Adams JM (2009) Phenotypic plasticity of leaf shape along a temperature gradient in Acer rubrum. PLoS ONE 4: e7653. 19893620 10.1371/journal.pone.0007653 PMC2764093

[13] Corney DPA, Clark JY, Tang HL, Wilkin P (2012) Automatic extraction of leaf characters from herbarium specimens. Taxon 61: 231–244.

[14] Royer DL, Wilf P (2006) Why do toothed leaves correlate with cold climates? Gas exchange at leaf margins provides new insights into a classic paleotemperature proxy. International Journal of Plant Sciences 167: 11–18.

[15] Feild TS, Sage TL, Czerniak C, Iles WJD (2005) Hydathodal leaf teeth of Chloranthus japonicus (Chloranthaceae) prevent guttation-induced ooding of the mesophyll. Plant, Cell & Environment 28: 1179–1190.

[16] Pigott CD (1997) Tilia. In: Cullen J, Alexander J, Brickell C, Edmondson J, Green P, et al., editors, The European Garden Flora, Cambridge: Cambridge University Press, volume V: 205–212.

[17] Clark JY (2009) Neural networks and cluster analysis for unsupervised classification of cultivated species of Tilia (Malvaceae). Botanical Journal of the Linnean Society 159: 300–314.

[18] Pigott CD (2012) Lime Trees and Basswoods: Biology, Ecology and Distribution of the Genus Tilia. Cambridge University Press. To appear.

[19] Cope JS, Corney DPA, Clark JY, Remagnino P, Wilkin P (2012) Plant species identification using digital morphometrics: A review. Expert Systems with Applications 39: 7562–7573.

[20] Belhumeur P, Chen D, Feiner S, Jacobs D, Kress W, et al (2008) Searching the world’s herbaria: A system for visual identification of plant species. In: Computer Vision - ECCV 2008, Springer. 116–129.

[21] Scotland RW, Wortley AH (2003) How many species of seed plants are there? Taxon 52: 101–104.

[22] Govaerts R (2001) How many species of seed plants are there? Taxon 50: 1085–1090.

[23] Thiers B (2011) Index Herbariorum: A global directory of public herbaria and associated staff, New York Botanical Garden’s Virtual Herbarium. http://sweetgum.nybg.org/ih/, accessed October 17th 2011..

[24] Viscosi V, Cardini A (2011) Leaf morphology, taxonomy and geometric morphometrics: A simplified protocol for beginners. PLoS ONE 6: e25630. 21991324 10.1371/journal.pone.0025630 PMC3184990

[25] Bylesjö M, Segura V, Soolanayakanahally R, Rae A, Trygg J, et al (2008) LAMINA: a tool for rapid quantification of leaf size and shape parameters. BMC Plant Biology 8: 82. 18647399 10.1186/1471-2229-8-82 PMC2500018

[26] Clark JY, Corney DPA, Tang HL (2012) Automated plant identification using artificial neural networks. In: 2012 IEEE Symposium on Computational Intelligence in Bioinformatics and Computational Biology (CIBCB). San Diego, California, USA: IEEE, p. 343–348.

